# The product of the *split ends *gene is required for the maintenance of positional information during *Drosophila *development

**DOI:** 10.1186/1471-213X-4-15

**Published:** 2004-12-13

**Authors:** Kimberly Mace, Antonio Tugores

**Affiliations:** 1 Surgical Research Lab; University of California, San Francisco, Box 1302, San Francisco, CA 94143-1302, USA; 2 Almirall Prodesfarma SA, Cardener 68, 08024 Barcelona, SPAIN; 3 Department of Biology, 0349. University of California, San Diego. 9500 Gilman Dr., La Jolla, CA 92093, USA

## Abstract

**Background:**

The *Drosophila split ends *(*spen*) gene encodes a large nuclear protein containing three RNP-type RNA binding motifs, and a conserved transcriptional co-repressor-interacting domain at the C-terminus. Genetic analyses indicate that *spen *interacts with pathways that regulate the function of Hox proteins, the response to various signaling cascades and cell cycle control. Although *spen *mutants affect only a small subset of morphological structures in embryos, it has been difficult to find a common theme in *spen *mutant structural alterations, or in the interactions of *spen *with known signaling pathways.

**Results:**

By generating clones of *spen *mutant cells in wing imaginal discs, we show that *spen *function is required for the correct formation and positioning of veins and mechanosensory bristles both on the anterior wing margin and on the notum, and for the maintenance of planar polarity. Wing vein phenotypic alterations are enhanced by mutations in the *crinkled *(*ck*) gene, encoding a non-conventional myosin, and correlate with an abnormal spatial expression of Delta, an early marker of vein formation in third instar wing imaginal discs. Positioning defects were also evident in the organization of the embryonic peripheral nervous system, accompanied by abnormal E-Cadherin expression in the epidermis.

**Conclusions:**

The data presented indicate that the role of *spen *is necessary to maintain the correct positioning of cells within a pre-specified domain throughout development. Its requirement for epithelial planar polarity, its interaction with *ck*, and the abnormal E-Cadherin expression associated with *spen *mutations suggest that *spen *exerts its function by interacting with basic cellular mechanisms required to maintain multicellular organization in metazoans. This role for *spen *may explain why mutations in this gene interact with the outcome of multiple signaling pathways.

## Background

The morphological complexity of metazoans is achieved through the regulation of multiple genes in an orchestrated spatial and temporal manner. One of these genes, *split ends *(*spen*), was initially identified in a screen for mutations affecting axonal outgrowth in the nervous system in *Drosophila *[1]. Additional mutations in *spen *were isolated in a screen for genetic modifiers of *Deformed *(*Dfd*) function. *Dfd *encodes a Hox transcription factor that specifies maxillary segment identity during development [2]. *spen *was subsequently found to enhance embryonic thoracic defects resulting from loss of function mutations in the Hox gene *Antennapedia *[3]. Other studies have found mutations in *Drosophila spen *as modifiers of mutations in components of Ras/MAP kinase pathways, including Raf kinase [4], kinase suppressor of Ras [5], loss of function mutations in the gene encoding the protein tyrosine phosphatase Corkscrew [6], and in the ETS family transcription factor, Aop/Yan [7,8]. Mutations in the *spen *gene have also been identified as enhancers of gain of function phenotypes caused by overexpression of E2F or Cyclin E in eye cells [9,10], both of which are required for progression through the S phase of the cell cycle, as well as *Dacapo*, a cyclin dependent kinase inhibitor [9]. Overexpression of Spen may interfere with Notch signaling during the development of adult external sensory organs [11], and *spen *function is required for the maternal expression of the Notch pathway transcription factor encoded by *Suppressor of Hairless (Su(H)) *[12]. Recent evidence also suggests that *spen *may participate in the transduction of the Wingless (Wg) signal within a subset of cells in the wing imaginal disc [13].

The Spen protein is ubiquitously expressed throughout embryogenesis. Differential splicing of *spen *results in isoforms encoding at least two proteins of ~5500 amino acids containing three tandem RNP-type RNA binding domains and a SPOC (Spen Paralogous and Orthologous C terminal) domain at the carboxy terminus [3]. These domains are highly conserved in both the mouse and human orthologs, called Msx-2 Interacting Nuclear Target (MINT) and SMRT/HDAC1 Associated Repressor Protein (SHARP), respectively. There is increasing evidence indicating that Spen-related polypeptides play a role in transcriptional repression. MINT may participate in bone development by binding to the *osteocalcin *promoter, via its RNP motifs, and repressing transcription in a binding complex with the homeodomain protein Msx-2 [14]. The interaction between SHARP and Silencing Mediator for Retinoid and Thyroid-hormone receptors (SMRT) can lead to the recruitment of histone deacetylase complexes through the conserved SPOC domain [15,16]. Both SHARP and MINT have also been proposed as negative regulators of the Notch signaling pathway in mammals. SHARP has been shown to bind directly to RBP-Jκ and repress the HES-1 promoter in an HDAC-dependent manner [17]. Although deletion of MINT coding sequences in mice results in embryonic lethality around E 14.5 due to multiple abnormalities, the analysis of hematopoiesis derived from MINT^-/- ^precursors reveals a defect in B cell development that could be attributed to defects in Notch signaling [18].

Despite the sum of genetic and biochemical evidence, a selective role for Spen-like proteins in a particular pathway in mammals or *Drosophila *remains unclear. Because wing development is a well characterized system for the study of primary pattern formation, diverse signaling pathways, and cell cycle control [19,20], we have used mitotic recombination in the wing disc to analyze *spen *mutant mosaics. An additional advantage is that, because wings are not essential for adult viability, the study of a large number of specimens is possible. In this study, we show that the function of *spen *is necessary for the maintenance of planar polarity, and for the correct formation and positioning of veins and mechanosensory bristles on the anterior wing margin and the notum. Alterations in vein formation in *spen *clones correlate with abnormal spatial expression of Delta, an early marker of vein formation in third instar wing imaginal discs. All wing phenotypes are enhanced in a *crinkled *(*ck*) mutant background, a gene encoding the non-canonical myosin VIIa. The abnormal position of sensory organs is also observed during embryonic PNS development. In contrast with previous reports, we show that *spen *is not essential for the determination of specific cell fates nor for cell survival, nor is it directly required for the outcome of the Ras, Notch, and Wingless signaling pathways. Based on our observations, we propose that *spen *is required for cells to maintain positional identity and tissue cohesiveness during the organized growth of wing and notum epithelia.

## Results

### *spen *function is essential throughout development

*spen *has been shown to be involved in many processes during embryonic development. Mutant modifier and gain of function screens indicate that *spen *participates in a variety of developmental processes in *Drosophila*, including Hox gene function [2,3], cell cycle control [9,10] and the modulation of signal transduction pathways such as Ras [4-8], Notch [11,12], and Wingless [13]. Given these varied interactions, it is unclear how *spen *functions through a common mechanism of action. To better understand a role for *spen*, we have generated genetic mosaics in adult tissues by using *Flp1*-mediated mitotic recombination [21].

The *spen*^*poc*361 ^and *spen*^*poc*231 ^mutant alleles have been previously described [3]. Although not molecularly characterized, there is evidence indicating that *spen*^*poc*361 ^is a null allele. First, in maternal and zygotic *spen*^*poc*361 ^mutant embryos, Spen protein cannot be detected with a polyclonal antibody raised against the region encoding amino acids 3203–3714 [[3], and not shown], suggesting that this mutant is either a protein null or that it is truncated before this region. Second, the *spen*^*poc*361 ^allele displays nearly the same strength as the TE21A deficiency, which deletes the entire locus [3].

Both *spen *alleles were recombined onto chromosomes containing an FRT sequence at 40A [3], and were subjected to heat shock driven Flp1-mediated mitotic recombination with a *M(2)*^36*F *^*FRT*^40*A *^chromosome. Heat shocks delivered at different times during larval and pupal development rendered few escapers (<1%), which presented with little or no mosaicism as revealed by the absence of the *M *dominant marker (not shown), indicating that the function of *spen *is essential during all stages of development.

### *spen *mutant mosaics affect vein morphology and planar polarity in adult wings

**Figure 1:**
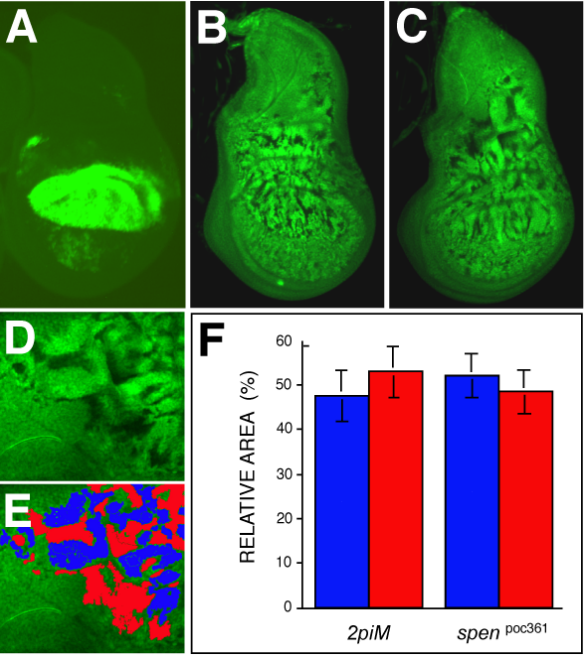
**Generation of *spen *mutant clones in wing imaginal discs**. (A) Expression of a UAS-GFP transgene driven by MS1096 GAL4 in third instar wing imaginal discs. As described previously [22], the transgene is expressed mainly in the dorsal wing pouch, with weaker expression in the ventral side and in the prospective notum. (B, C) Virgin females with the genotype {*MS1096 GAL4; 40 2piM FRT*^40*A*^}, or {*MS1096 GAL4; spen*^*poc*361 ^*FRT*^40*A*^*/ ln (2LR) CyO*}, were crossed to {w*; GFP FRT^40*A*^*; UAS-Flp1/TM6b*} males. Shown are wing imaginal discs isolated from the resulting third instar larvae with the genotypes {*MS1096 GAL4/+; 40 2 piM FRT*^40*A*^*/GFP FRT*^40*A*^*; UAS Flp1/ +*} (B), and {*MS1096 GAL4/+; spen*^*poc*361 ^*FRT*^40*A*^*/GFP FRT*^40*A*^*; UAS-Flp1/ +*}(C). Green fluorescence reveals either compound heterozygous cells (not subjected to mitotic recombination) or GFP homozygotes (brightest), while dark spots indicate *2 piM *(B) or *spen*^*poc*361 ^(C) homozygous clones. Dorsal is up and anterior is to the left. To know the relative area covered by either *2 piM *or *spen*^*poc*361 ^homozygous clones versus *wt *clones, regions of equal intensity within the images were artificially colored in Adobe Photoshop using the Paint Bucket tool (D, E). Colors corresponding to mutant (red) or *wt *(blue) areas were extruded independently, and the total number of pixels contained within the regions of interest were calculated using the Kodak 1D Image Analysis Software (Eastman Kodak Company, Rochester, NY). The results are represented as the fraction covered by each genotype for each cross in a total of three discs for the crosses involving *2 piM*, and 5 discs for *spen*^*poc*361 ^(F).

Because the studies described above did not provide any information about the nature of this lethality, mosaics were generated through expression of the Flp1 recombinase in wing imaginal discs, where *spen *mRNA is expressed ubiquitously as revealed by RNA *in situ *hybridization (not shown). Using the *MS1096-GAL4 *driver [[22], Figure 1A], the expression of a *UAS-Flp1 *transgene [23] induced mitotic recombination in wing discs between the *FRT *chromosome bearing a *spen *mutant allele and an *FRT *chromosome containing a ubiquitously expressed *GFP *transgene on 2L. The homozygous viable *2piM FRT*^40*A *^chromosome [21] was used as a control in all crosses. Initially, to avoid non-specific interference of additional mutations in the analysis (see below), clones were not phenotypically marked in adults, although their formation could be followed in discs by looking at the expression of the *GFP *transgene. In order to prevent the evaluation of non-specific abnormalities caused by the *MS1096-GAL4 *insertion on the X chromosome of hemizygous males, only heterozygous females were analyzed.

Using this approach, clones were generated on the dorsal side of the wing pouch, haltere imaginal discs, and to a minor extent on the ventral side of the wing pouch and on the prospective scutellum (Figure 1A-C). In third instar imaginal discs, the relative area covered by *spen *mutant cells was comparable in size to that obtained in the control crosses (Figure 1F), indicating that loss of function of the *spen *gene did not appear to autonomously affect cell viability during growth of the disc.

**Figure 2:**
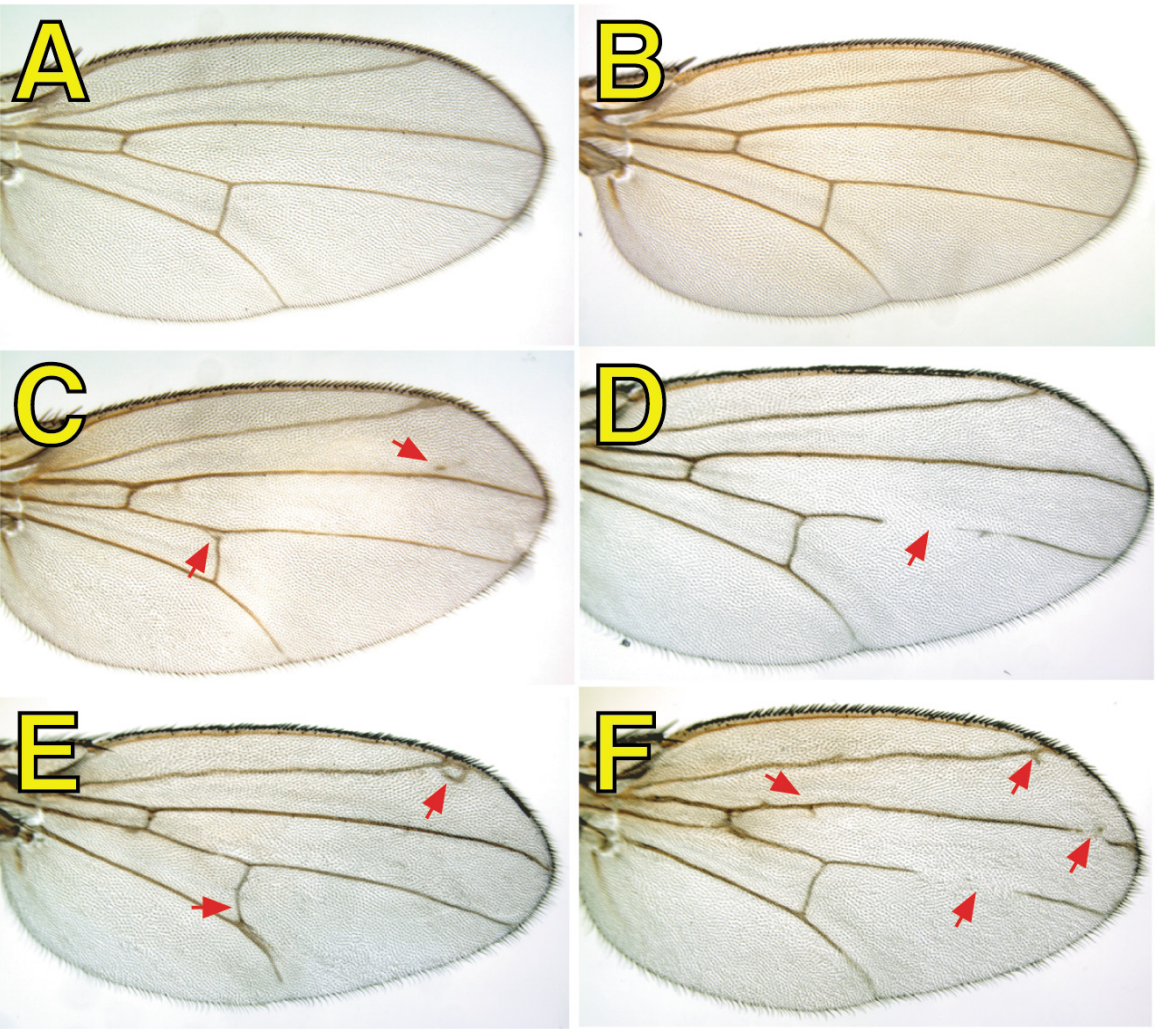
**The presence of *spen *mutant clones affects wing vein morphology**. Virgin females with the genotype {*MS1096 GAL4; 40 2piM FRT*^40*A*^}, {*MS1096 GAL4; spen*^*poc*231 ^*FRT*^40*A*^*/ ln (2LR) CyO*}, or {*MS1096 GAL4; spen*^*poc*361 ^*FRT*^40*A*^*/ ln (2LR) CyO*}, were crossed to {w*; GFP FRT^40*A*^*; UAS-Flp1/TM6b*} males, and adult wings were isolated from progeny females with the following genotypes: {*MS1096 GAL4/+; 40 2 piM FRT*^40*A*^*/GFP FRT*^40*A*^*; UAS Flp1/ +*} (A), {*MS1096 GAL4/+; spen*^*poc*361 ^*FRT*^40*A*^*/ GFP FRT 40A; TM6b/ +*} (B), {*MS1096 GAL4/+; spen*^*poc*231 ^*FRT*^40*A*^*/ GFP FRT*^40*A*^*; UAS-Flp1/ +*} (C, D), and {*MS1096 GAL4/+; spen*^*poc*361 ^*FRT*^40*A*^*/ GFP FRT*^40*A*^*; UAS-Flp1/ +*} (E, F). Arrows indicate gain (C, E, F), loss (D, F), or misplacement (E) of vein material.

**Figure 3:**
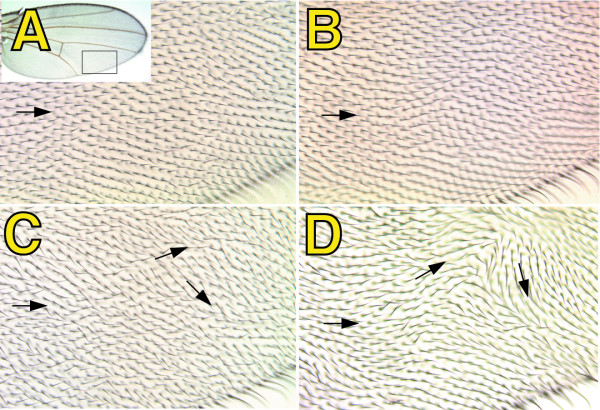
***spen *mutations affect wing hair polarity**. Crosses were performed as described in Figure 1. Shown are details (see inset in A) of adult wings isolated from progeny females with the genotypes: {*MS1096 GAL4/ +; 40 2piM FRT*^40*A*^*/ GFP FRT*^40*A*^*; UAS-Flp1/ +*} (A), {*MS1096 GAL4/ +; spen*^*poc*361 ^*FRT*^40*A*^*/ GFP FRT*^40*A*^*; TM6b/ +*} (B), {*MS1096 GAL4/ +; spen*^*poc*231 ^*FRT*^40*A*^*/GFP FRT*^40*A*^*; UAS-Flp1/ +*} (C), and {*MS1096 GAL4/*+*; spen*^*poc*361 ^*FRT*^40*A*^*/ GFP FRT*^40*A*^*; UAS-Flp1/ +*} (D). Arrows indicate the direction of the bristles.

**Figure 4:**
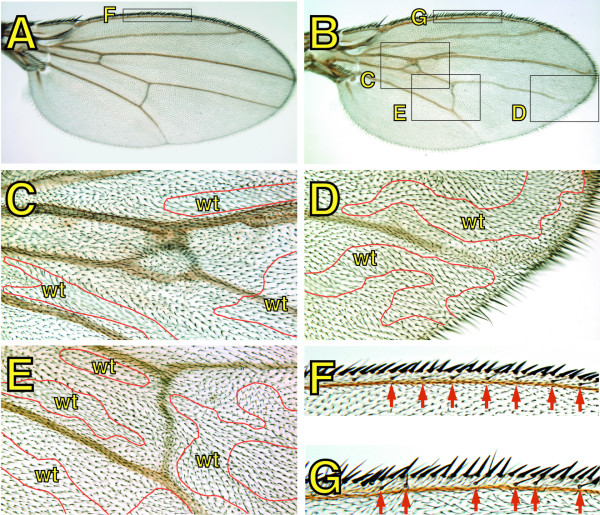
**Incorrect positioning of wing elements in mosaic *spen *mutant wings is enhanced by mutations in the *crinkled *(*ck*) gene**. Virgin females with the genotype {*MS1096 GAL4; ck FRT*^40*A*^*/ ln (2LR) CyO *}, or {*MS1096 GAL4; spen*^*poc*361^*ck FRT*^40*A*^*/ ln (2LR) CyO *}, were crossed to {w*; GFP FRT^40*A*^*; UAS-Flp1/ TM6b*} males, and adult wings were isolated from progeny females with the following genotypes: {*MS1096 GAL4/+; ck FRT*^40*A*^*/ GFP FRT*^40*A*^*; UAS-Flp1/ +*} (A, F), {*MS1096 GAL4/+; spen*^*poc*361 ^*ck FRT*^40*A*^*/ GFP FRT*^40*A*^*; TM6b/ +*} (B-E, G). Red lines separate either wt or compound heterozygous cells (indicated as wt) from *spen *mutant cells.

The generation of *spen *mutant mosaics during wing development caused phenotypic abnormalities that included both the formation of ectopic patches of vein material and the loss of vein material (mostly distally), as well as subtle mis-localization of both longitudinal and cross veins, frequently accompanied by thickening of the veins (Figure 2). The ectopic vein material was always observed around veins, and was never detected in the middle of intervein regions. Additional abnormalities included the disruption of cell polarity, as evidenced by the abnormal orientation of wing blade trichomes (Figure 3) and the mis-placement of bristles at the wing margin (see below on Figure 4), whereas their morphology appeared normal. No disruptions in any of the main axes of the wing (A/P, D/V and P/D) were observed in wings containing *spen *mutant clones.

All phenotypes were more penetrant when the *spen*^*poc*361 ^allele was used instead of the *spen*^*poc*231^, confirming previous results indicating that *spen*^*poc*361 ^represents a stronger allele [2,3]. None of the phenotypes described above were observed when a control chromosome was subjected to mitotic recombination (Figures 2A, and 3A), or when either the driver (*MS1096-GAL4*), or the *UAS-Flp1 *transgene, were independently present in a *spen *heterozygous background (Figures 2B, 3B, and not shown).

### Morphological alterations in mosaic *spen *mutant wings are enhanced by mutations in *crinkled *(*ck*)

To determine whether the phenotypes observed in *spen *mosaic mutant wings were cell autonomous, *spen *mutant chromosomes were marked with *crinkled *(*ck*), a commonly used recessive marker on 2L. While the presence of *ck *homozygous clones had no effect on wing vein patterning and morphology (Figure 4A), the presence of *ck *on the *spen *mutant chromosome markedly enhanced the severity of the phenotypes previously observed with *spen *mutants alone (Figure 4B). Likewise, we observed an excess (Figure 4C) or absence (Figure 4D) of vein material, and misplacement of both longitudinal and cross veins (Figure 4E). In most cases, these abnormalities correlated with the presence of the *ck *marker phenotype. However, there were cases in which clones on the dorsal side affected vein morphology on the ventral side and *vice versa*. Therefore, the phenotypic effects caused by *spen *mutations are not exclusively cell autonomous, although it appears autonomous in cells within a given cell layer.

A "misplacement" effect was also observed at the wing margin, most frequently affecting the dorsal bristles. In a normal wing, the bristles are evenly spaced in a row along the dorsal side of the anterior wing margin (Figure 4). In the presence of *spen *mutant clones, the spacing between these bristles was altered (Figure 4G). Additionally, a mis-alignment and occasional tufting of the thick trichomes that are found along the wing margin was also observed. Again, these phenotypes were not exclusively cell autonomous, as was the case with aberrations in wing vein morphology. Alternatively, it is plausible that *spen *mutations alter the phenotypic manifestation of *ck *mutants, therefore leading to an incorrect conclusion about the autonomy of the *spen *mutations.

### *spen *mutant clones disrupt the expression of Delta and Cut in wing imaginal discs

**Figure 5:**
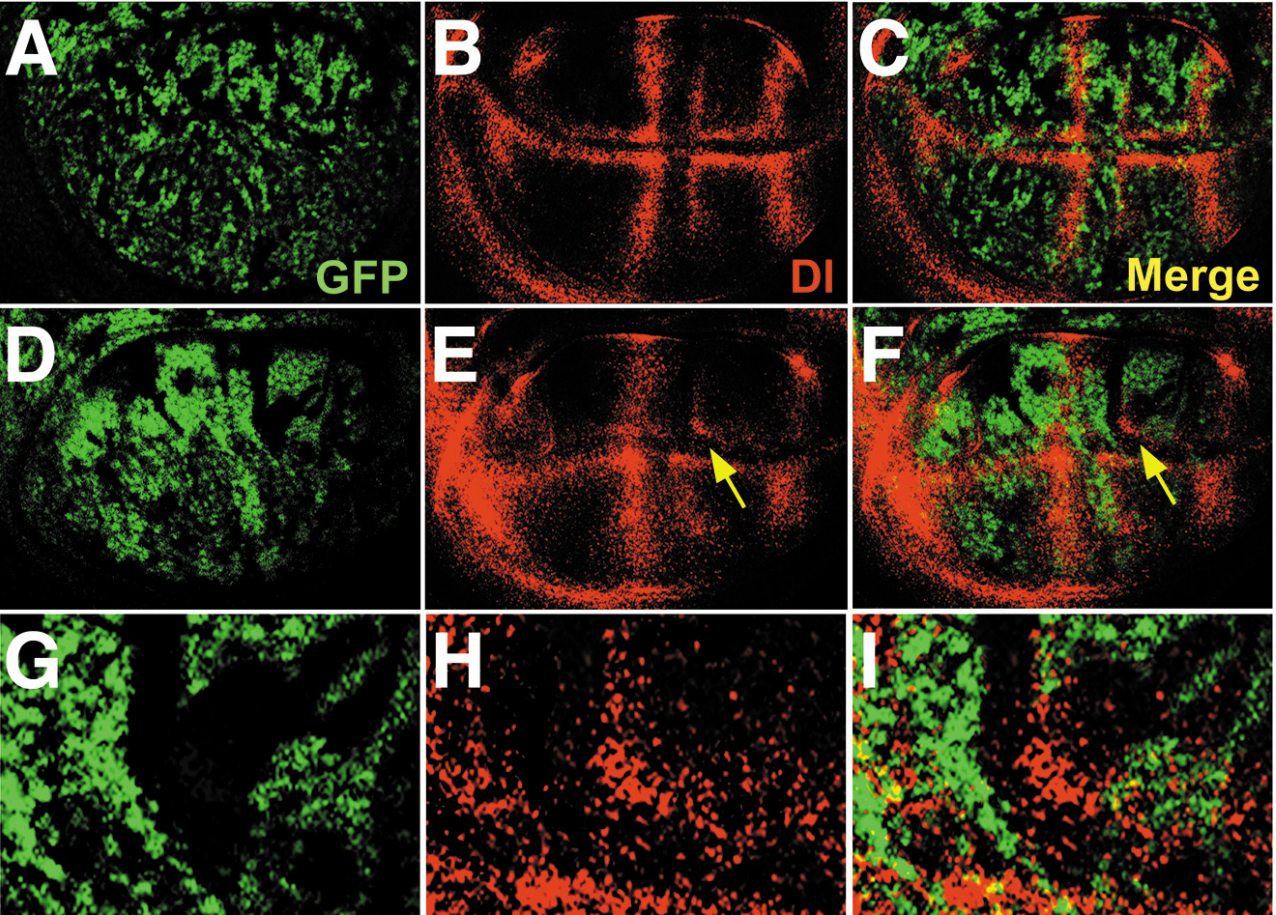
**Delta protein expression is abnormally distributed in the presence of *spen *mutant clones**. Crosses were performed as described in Figure 1, and third instar imaginal disks were isolated from progeny female larvae with the following genotypes: {*MS1096 GAL4/+; 40 2piM FRT*^40*A*^*/ GFP FRT*^40*A*^*; UAS-Flp1/ +*} (A-C), and {*MS1096 GAL4/ +; spen*^*poc*361 ^*FRT*^40*A*^*/ GFP FRT*^40*A*^*; UAS-Flp1/ +*} (D-I). Delta expression (in red) was detected in by using a mouse anti-Delta MAb followed by a Cy3-conjugated anti mouse antibody. The area indicated by the arrows in E and F is shown at higher magnification in G to I. Dorsal is up and anterior is to the left.

Veins are generated in specific domains within the wing field and require the action of early patterning genes that establish basic positional values composing the main axes (D/V, A/P). This process is followed by the initiation of vein formation, and finally, vein differentiation [19]. The phenotypes observed upon generation of *spen *mutant clones in wing imaginal discs are consistent with a role for *spen *at later stages when vein formation takes place, as the establishment of compartment boundaries appears unaffected (Figure 2). One gene product that is involved in early vein patterning and differentiation is Delta (Dl), which participates in delimiting vein boundaries along prospective vein forming domains through lateral inhibition [19]. In third instar wing imaginal discs, expression of Dl correlates with the prospective L3, L4, and L5 veins (see Figure 5B).

Dl protein expression was normal in third instar wing discs containing homozygous clones for both the *2 piM*, and the *GFP FRT *marker chromosomes, which confer nuclear GFP expression (Figure 5A-C). In the presence of *spen *mutant clones, Dl expression was inconsistently abnormal: In some cases we observed ectopic expression of Dl within *spen *mutant clones, although this was not the norm. Similarly, Dl expression could be absent from normal regions adjacent to *spen *mutant cells, suggesting a non-autonomous effect (Figure 5D-I). Most frequently, however, the abnormal expression of Dl within a *spen *clone was consistent with a shift or misplacement of Dl-expressing cells. As shown in Figure 5 (Panels E, F and H, I), Dl-positive cells are located away from the position where they are expected, at the intersection of the prospective L4 vein at the wing margin.

**Figure 6:**
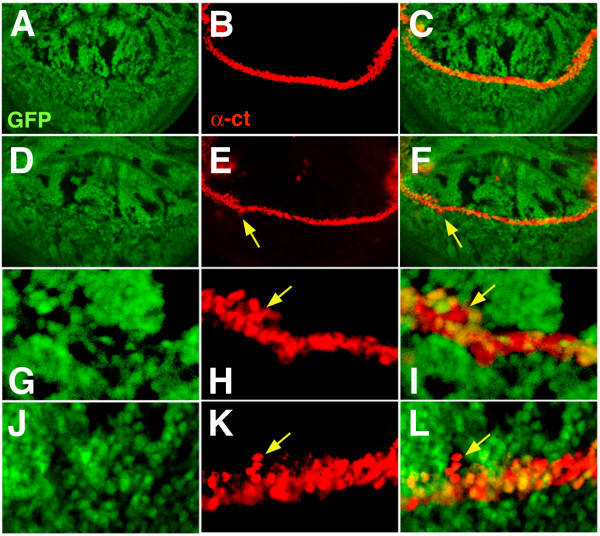
**Analysis of Cut protein expression in the presence of *spen *mutant clones**. Crosses were performed as described in Figure 1, and third instar imaginal disks were isolated from progeny female larvae with the following genotypes: {*MS1096 GAL4/+; 40 2piM FRT*^40*A*^*/ GFP FRT*^40*A*^*; UAS-Flp1/ +*} (A-C), and {*MS1096 GAL4/ +; spen*^*poc*361 ^*FRT*^40*A*^*/ GFP FRT*^40*A*^*; UAS-Flp1/ +*} (D-L). Cut expression (in red) was detected with an anti-Cut MAb as described in Figure 4 for Dl, and the absence of GFP (in green) defines mutant cells as explained on Figure 4. G to H shows a magnification of the spot indicated by an arrow on E and F. Note that the Cut protein is present in *spen *mutant cells. Panels J to L show the margin of another disc not shown in the figure. The arrow indicates a group of heterozygous cells that are away from the margin, surrounded by a group on *spen *mutant cells. Dorsal is up and anterior is to the left.

One of the genes whose expression delineates the D/V boundary at the wing margin beginning at mid to late third instar is the homeodomain transcription factor Cut (Ct) [24,25]. The analysis of Ct expression is of particular interest for our study because both the *N *and *wg *signaling pathways cooperate to maintain its expression at the margin [26,27], and both signaling pathways have been reported to be affected by *spen *mutations [11-13]. As shown (Figure 6), the expression of Ct was not subjected to major alterations at the wing margin in the presence of *spen *mutant clones. Occasionally, Ct expression was broader, or detected in cells a few cell diameters away from the margin (Figure 6G-L), coinciding with the presence of *spen *mutant cells. This observation is consistent with our previous results showing abnormal spatial expression of Dl, which is in turn required to restrict Ct expression to the margin [27].

### The organization of the PNS is abnormal in both adult *spen *mutant clones and maternal and zygotic *spen *mutant embryos

It was unclear whether the effect of *spen *mutant clones on the spacing of dorsal sensory bristles at the anterior wing margin was due to abnormal positioning or to a defect in the correct specification of sensory organ precursors. To determine if this effect could be generalized to other adult structures, expression of the Flp1 recombinase was directed to the anterior compartment of the wing disc with a *dpp*^*DISC*^*-GAL4 *driver [28]. Although in this case the clones were not marked in adults, analysis of GFP expression in third instar imaginal discs showed that *spen *mutant clones were generated throughout the anterior compartment of the wing disc, including most of the prospective notum (data not shown).

**Figure 7:**
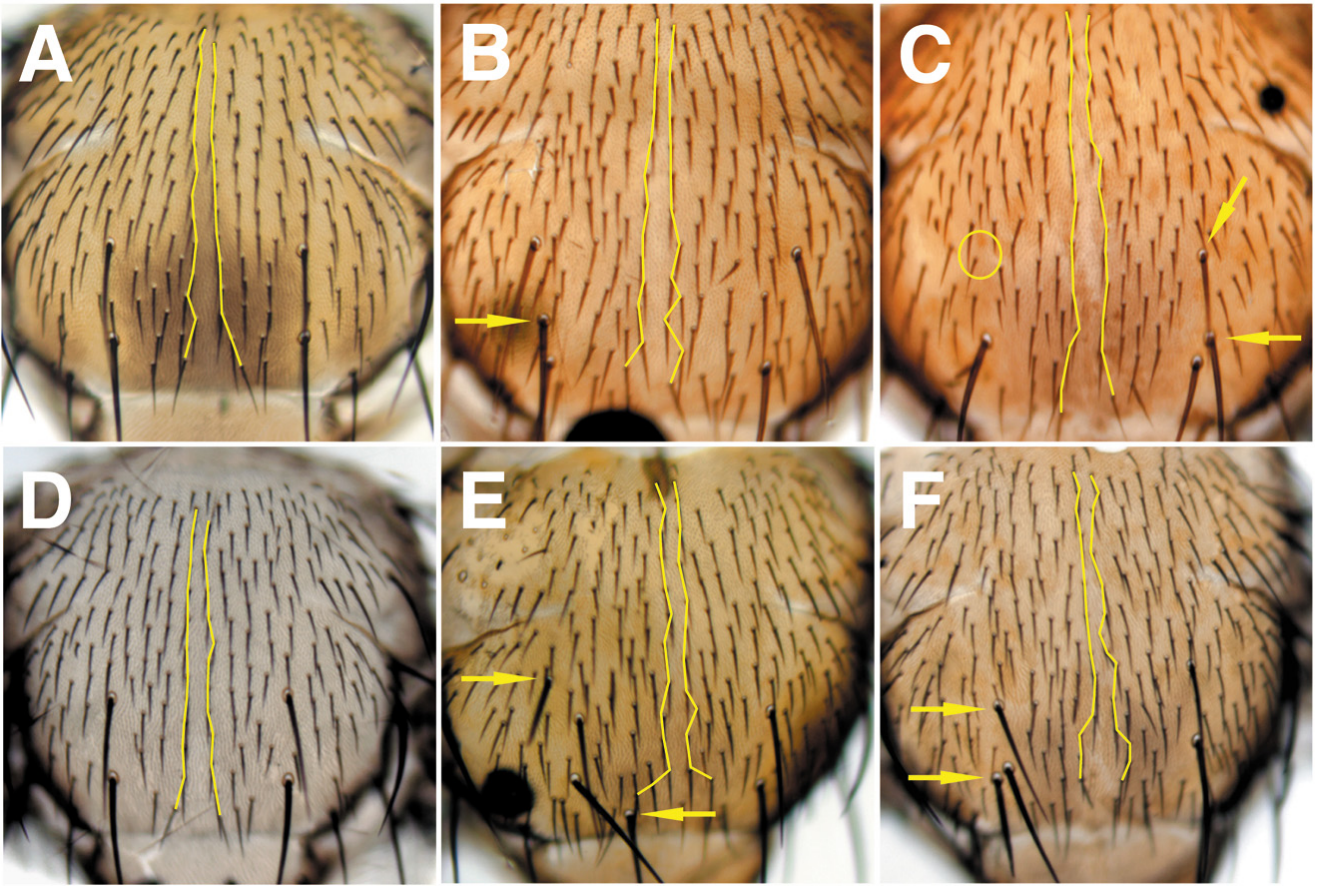
**Sensory bristle number and position is abnormal in notums containing *spen *mutant clones**. Virgin females with the genotype {*spen*^*poc*231 ^*FRT*^40*A*^*/ ln (2LR) CyO; dpp*^*DISK *^*GAL4/ TM6b*} or {*spen*^*poc*361 ^*FRT*^40*A*^*/ ln (2LR) CyO; dpp*^*DISK *^*GAL4/ TM6b*}, were crossed to {w*; GFP FRT^40*A*^*; UAS-Flp1/ TM6b*} males, and adult notums were isolated from progeny females (A-C), or males (D-F) with the following genotypes: {*spen*^*poc*361 ^*FRT*^40*A*^*/ GFP FRT*^40*A*^*; dpp*^*DISK *^*GAL4/ TM6b*} (A, D), {*spen*^*poc*231 ^*FRT*^40*A*^*/ GFP FRT*^40*A*^*; dpp*^*DISK *^*GAL4/ UAS-Flp1*} (B, E), and {*spen*^*poc*361 ^*FRT*^40*A*^*/ GFP FRT*^40*A*^*; dpp*^*DISK *^*GAL4/ UAS-Flp1*} (C, F). Lines delineate the bristles at the dorsal midline. Empty circles indicate loss of bristles (in C), and arrows indicate either gain, or abnormal location of macrochaetae (B, C, E, and F).

The generation of large *spen *mutant clones in the notum severely affected the final adult pattern of both the macro and microchaetae (Figure 7). This patterning defect was easily observed in the alignment of microchaetae along the dorsal midline, and the overall phenotype was more penetrant in males than in females. The number and position of macrochaetae were also affected, and the observed defects included their loss (Figure 7C), gain (Figure 7B,C,E, and 7F), and abnormal positioning (Figure 7B,C,E,F). In each case, these abnormalities were never associated with the appearance of double trichogens (bristles) or thormogens (sockets), which would indicate a defect in the specification of particular cell fates during the formation of the external sensory organs.

**Figure 8:**
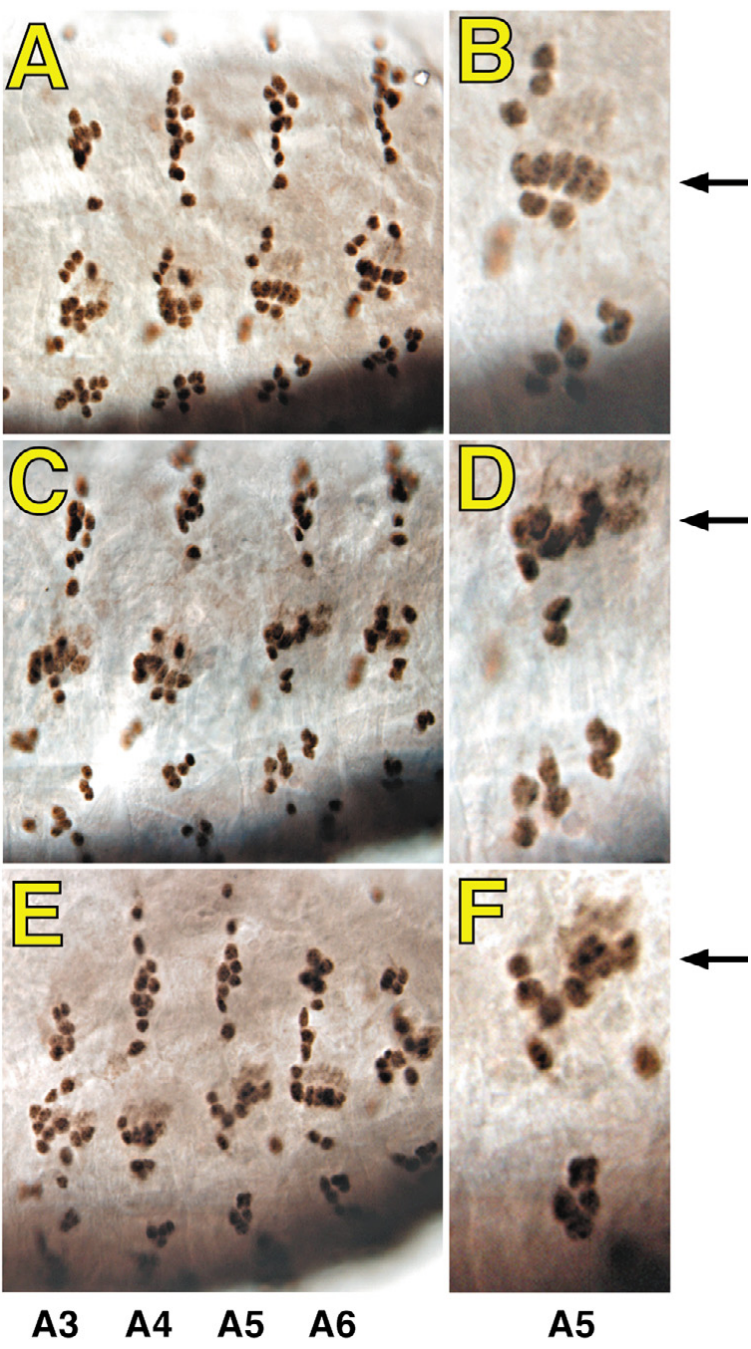
**Abnormal positioning of PNS neurons in *spen *maternal and zygotic mutant embryos**. Maternal and zygotic *spen *mutant embryos were obtained as described in Materials and Methods, and at stage 14–15 were stained for the neuron specific marker Elav, together with an anti β-galactosidase antiserum to reveal the presence of the *CyO*, *wg-lacZ *balancer in heterozygotes (not shown), followed by biotinylated secondary antibodies, and streptavidin conjugated horse radish peroxidase. Brown staining reveals the nuclei of PNS neurons in *wt *(A, B), maternal and zygotic *spen*^231 ^(C, D), or *spen*^361 ^(E, F) embryos.

Previous reports have linked *spen *to the organization of the embryonic peripheral and central nervous systems [1,8,12]. In agreement with these studies, and with our findings in adults, we observed an abnormal distribution of neurons in *spen *maternal and zygotic embryos, as evidenced by immunodetection of the pan neural marker Elav (Figure 8). Similar to the previously described phenotypes, they had variable penetrance with a change in the number of Elav positive cells (either more of fewer) not consistently observed.

### Abnormal PNS distribution correlates with altered epidermal expression of E-cadherin in spen mutant embryos

Peripheral neurogenesis starts at the epidermis, where sensory organ precursors are specified, and give rise to sensory organ cells through carefully polarized cell divisions. Aside from these divisions, additional cell types might be recruited from the epidermis to take part in the formation of internal sensory organs [reviewed in [29]]. Increased epithelial activity at the sites of sensory organ formation may reveal defects in cell adhesion, such as those caused by mutations in the E-Cadherin mutant *shotgun *(*shg*), whose loss of function results in holes in the epithelium. These holes, which later appear in the cuticle, presumably arise from a failure to re-establish a *status quo *at sites of high morphogenetic activity [30].

**Figure 9:**
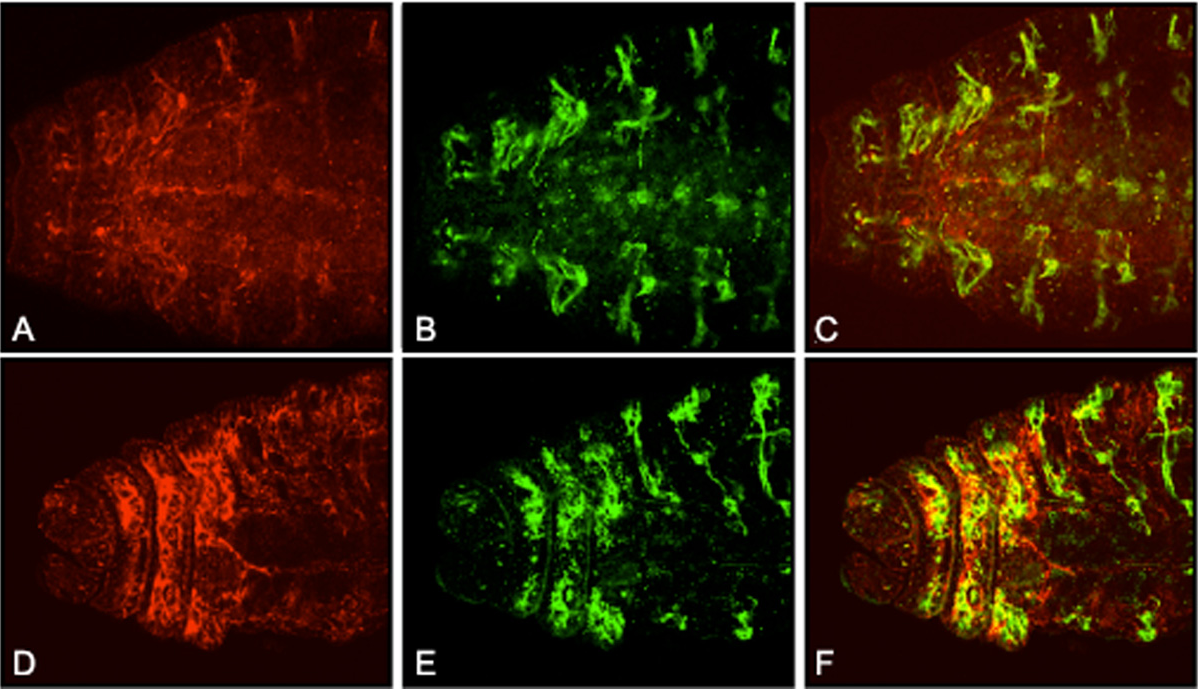
**Increased epidermal expression of E-cadherin correlates with abnormal positioning of embryonic PNS neurons**. Stage 16 wild type embryos (A-C) or maternal and zygotic *spen *mutant embryos (D-F) were generated as described in Materials and Methods. The expression of the PNS neuronal marker 22C10 (green) and E-cadherin (red), were detected with specific MAbs followed by fluorescein conjugated anti-mouse antiserum and a Cy3 conjugated anti rat as described earlier in Figure 4, and in the Material and Methods section.

The fact that *spen *mutant embryos die at the end of embryogenesis with sclerotic patches and holes in their epidermis in the ventrolateral thorax and lateral abdomen [[3]; K. Mace, J. Pearson, W. McGinnis, submitted], together with the evidence that the embryonic PNS is disorganized in these embryos, may suggest a defect in cell adhesion at these sites. As shown in Figure 9, embryos lacking *spen *function display abnormal PNS neuron positioning and morphology compared to wild type as revealed by 22C10 staining (compare Figures 9A and 9D). The surrounding epidermal cells show a dramatic upregulation of E-cadherin (compare Figures 9B and 9E). The placement of these abnormal neurons is precisely within this field of epidermal cells (see merge, Figure 9F) that have been shown to be undergoing a wound response due to a failure of epidermal epithelial integrity [K Mace, J Pearson, W McGinnis, submitted].

Because E-cadherin is an important intercellular adhesion molecule, we wanted to test whether the defects seen in the developing adult wing were also associated with changes in its expression. Additionally, we assayed the expression of Crumbs, another component of the adherens junction, and β-tubulin, a marker of cell polarity. None of these proteins showed any detectable changes in expression or localization within *spen *mutant clones in the developing wing disc (not shown). It is possible that, because the wing disc epithelium is not subject to the same level of stress as the embryonic epidermis at the sites of internal sensory organ formation, upregulation of E-Cadherin expression is specific to the embryonic phenotype, and does not occur in wing discs. Therefore, it remains unclear how *spen *could affect, if at all, cell adhesion in this tissue.

## Discussion

### *spen *mutant cells are viable

The generation of large *spen *mutant clones in adults, using heat-shock driven expression of FLP recombinase in a *Minute *background indicated that *spen *function was essential during all stages of development. This result is consistent with the pleiotropic effects previously described for *spen*. However, when clones were generated specifically in wing imaginal discs, we observed that mutations in the *spen *gene did not affect the viability or the size of mutant cells during growth of the disc, as evidenced by normal cell size and number within the adult clones. These observations are intriguing given previous reports linking the function of *spen *to cell cycle progression [9,10]. In these studies, *spen *mutations were shown to enhance morphological abnormalities upon overexpression of wild type E2F [9] and Cyclin E [10], leading to the conclusion that *spen *has a negative regulatory role on these cell cycle regulators. If *spen *had a negative role in cell cycle progression in the wing imaginal disc, we would expect that *spen *mutant clones would be larger than their twin spots after mitotic recombination. However, other authors have observed that *spen *mutant clones are indeed smaller than their wild type counterparts [13]. If that were the case, we should expect that the *spen *mutant cells would increase their size, in order to offset a decrease in cell division rates, as it is the case for E2F mutants [20]. However, as stated above, both cell size and total area coverage were similar between *spen *mutant and wild type cells. This observation is in agreement with our observations in *spen *maternal and zygotic mutant embryos, which do not show differences in cell cycle progression *versus *wt embryos as assessed by 8-Bromo deoxy-Uridine incorporation, *string *transcript expression, or Proliferating Cell Nuclear Antigen (PCNA) protein expression (data not shown). Alternatively, there is no evidence of increased cell death in *spen *mutant cells as revealed by Acridine Orange staining, or *reaper *mRNA expression (data not shown). Therefore, we conclude that the function of the *spen *gene *per se *is not essential for cellular viability and normal progression through the cell cycle.

### *spen *and Ras signaling

Recently, there has been increasing experimental evidence suggesting that the product of the *spen *gene might be an integral component of the Ras signaling cascade. Such an interaction has been found in the search for genes interacting with a viable allele of *corkscrew *[6], and in gain of function screens utilizing the overexpression of components of the Ras pathway during eye development. These include activated Raf [4], *kinase supressor of ras *(*ksr*) [5], and a constitutive repressor form of Anterior Open (Aop) /Yan [7]. Wing vein formation in *Drosophila *provides an amenable system to analyze mutations affecting the MAPK pathway, as it depends on the function of the EGF receptor (DER), as well as other genes encoding components of the pathway, such as *Star (S)*, *rhomboid (rho)*, and the DER ligand, *vein (vn) *[19,31]. While loss of *rho *and *S *function result in non-autonomous loss of vein phenotypes, gain of function of *DER *and *rho *have the opposite effect [19,31,32].

If the product of the *spen *gene were an integral component of the Ras pathway, we would expect that the loss of function of *spen *would generate similar phenotypes as those obtained with other genes acting in the DER pathway. However, this was not the case: *spen *mutant clones generated during wing development showed both indiscriminate loss and gain of vein material, resulting in a vein phenotype that could not be directly correlated with any mutant in the Ras pathway known to date. Similarly, the phenotypes observed in *spen *mutant embryos do not clearly correlate with mutations in components of the Ras signaling pathway. For instance, the expression of orthodenticle (*otd*) mRNA at the ventral midline, which is dependent on DER function, and is abnormal in mutants that are defective in this signaling pathway [33], was indistinguishable between *spen *maternal and zygotic mutant embryos and wild type embryos (data not shown). Thus, our data do not support a direct correlation between the loss of *spen *function and a specific defect in DER/Ras-dependent signaling during embryogenesis or during imaginal disc development.

### *spen *and Notch signaling

A relationship between *spen *and Notch (N) function has been previously suggested, where the former appears to be necessary to maintain the embryonic expression of *Suppressor of Hairless *(*Su(H)*) [12] a downstream effector of N [34]. The observation that the gain of function of *spen *may also interact with N signaling [11] further strengthens the relationship between N and *spen *function. Additionally, there is supporting biochemical evidence indicating that both the human and murine orthologs of Spen, interact with RBP-Jκ/CBF-1, a mammalian ortholog of Su(H) [17,18]. The interaction of both SHARP and MINT with RBP-Jκ/CBF-1 prevents the interaction of the latter with the intracellular fragment of activated N, thus suggesting that both SHARP and MINT are negative regulators of N signaling in mammals. On the other hand, inactivation of the murine MINT gene does not clearly reflect a defect in N signaling. Loss of function of MINT in hematopoietic precursors, revealed that splenic B cells differentiated more efficiently toward the marginal zone than to the follicular type. This phenotype, attributed by the authors to a defect in N signaling, is in conflict with the fact that the election of T *versus *B cell fates in the lymphoid lineage, also dependent on N signaling, appears unaffected [18]. Indeed, variations in the numbers of follicular and marginal zone B cells, as reported for the MINT-deficient B cells, may also be attributed to migration defects leading to their abnormal distribution within the spleen [35]. Thus, it seems that a specific role for MINT in the mammalian N pathway has not been clearly defined yet.

The displacement of veins observed in adult wings in the presence of *spen *mutant clones, frequently accompanied by widening of the veins, is a phenotype consistent with defects in the N signaling pathway [19]. Furthermore, this phenotype correlates with abnormal (diffuse and/or ectopic) expression of Delta (Dl) in third instar imaginal discs both in *spen *mutant clones, and in cells adjacent to these clones. However, the role of the N signaling pathway has been well established as crucial for the determination of cell types during the development of external sensory organs. Defects in the N pathway alter sense organ cellular composition by affecting alternative cell fate decisions [36,37]. In our experimental system, the generation of *spen *mutant clones did not interfere with bristle formation *per se*, but with their spatial distribution. Both micro and macrochaetae were incorrectly positioned throughout the notum and, in some cases, chaetae were absent, but ectopic supernumerary bristles were also seen. Nevertheless, all external sensory organs appeared to have normal morphology, suggesting that there were no mis-specifications of cell fates, as it would be expected for N signaling defects. Additional evidence that supports this is that the expression of Cut at the wing margin, which is dependent upon both Su(H) and N function [26,27], was detected within *spen *mutant clones. Therefore, we conclude that the function of *spen *is not essential for N signaling in wing imaginal discs.

### *spen *and wg signaling

Recent evidence suggests that *spen *is required to transduce some aspects of the Wingless (Wg) signal in the wing imaginal disc, showing a requirement for *spen *for the expression of *senseless*, a downstream target of the Wg pathway [13]. The loss of Senseless in *spen *mutant clones would be predicted to lead to the absence of external sensory organs in adult wings [38], which is a phenotype that we did not observe with the alleles tested. Furthermore, we could not consistently correlate any of the abnormalities observed in adult wings containing *spen *mutant clones to known defects in the Wg signaling pathway. Clonal analysis in third instar imaginal discs did not reveal specific defects in the Wg pathway either: Wg signaling is required for Dl expression at the wing margin [27], and Dl expression was topologically affected but not absent in *spen *mutant clones. Taken together, it appears that *spen *function is required for one Wg signaling target (Senseless) [13], but not for others (Dl). These results, that could be explained by the differential penetrance of the different *spen *alleles used, do not seem to support a principal role for Spen in Wg signaling during wing imaginal disc development.

### A general role for Spen?

Based on previous reports and the data presented herein, the available evidence does not support a specific role for *spen *in any signaling pathway in particular. We would like to propose that the common theme that could best define *spen *function at the morphological level is that it appears necessary for the correct spatial organization of individual cells within a specific group during growth and development.

How could Spen instruct cells to maintain a specific position, without affecting their fate directly? A plausible explanation is that it could affect cell adhesion. In fact, in *spen *mutant embryos, the expression of E-cadherin was up-regulated at sites of high epithelial morphogenetic activity, generating a phenotype similar to the E-cadherin mutant embryos, as it has been observed in other studies [39]. It is plausible that the increase in E-cadherin expression is the result of a wound response to a defect in epithelial integrity, caused by *spen *mutations [K. Mace, J. Pearson, and W. McGinnis, submitted]. A defect in cell adhesion and/or cytoskeletal rearrangements could also explain specific aspects of the *spen *embryonic phenotype. The holes that result in abnormal cuticle deposition in the embryonic epidermis are due to a failure of epidermal epithelial integrity. These cells subsequently undergo a wound response at the end of embryogenesis. Additionally, some of the phenotypes resulting from the loss of *spen *are indeed similar to those seen in mutants for the gene encoding Daschous, a cadherin involved in cell adhesion [40]. However, blistering of the wings, a phenotype that is often found in cell adhesion mutants, was never observed in any of the *spen *mutant clones.

A role for Spen in cell adhesion and/or cytoskeletal rearrangements could also be inferred through its genetic interaction with *crinkled *(Myosin VIIA), and the planar cell polarity phenotype observed in mutant cells for *spen *in the wing blade. Myosin VIIA is associated with the cadherin-catenins complex and participates in the creation of a tension force between the actin cytoskeleton and adherens junctions, which is predicted to strengthen cell-cell adhesion [41]. Furthermore, *ck *acts downstream of *Drosophila Rho-associated kinase *(*Drok*), which links Frizzled-mediated planar cell polarity signaling to the actin cytoskeleton [42]. Myosin VIIA mutations have been described in vertebrates, including those causing the Usher syndrome in humans [43], the *shaker-1 *mutation in mice [44], and the *mariner *mutation in zebrafish [45]. Interestingly, these mutations, among other symptoms, cause splaying and abnormal distribution of sensory hair cells in the inner ear, leading to deafness in mice and humans, and mechanosensory defects in zebrafish. It seems plausible that *spen *may regulate the expression or function of components affecting the outcome of pathways involved in cytoskeletal rearrangements and epithelial planar polarity and, hence, affect cell positioning. However, a direct requirement for *spen *function in the Ck or Drok pathways is unlikely, since mutations in these genes result in different phenotypes than those observed in *spen *mutants.

**Figure 10:**
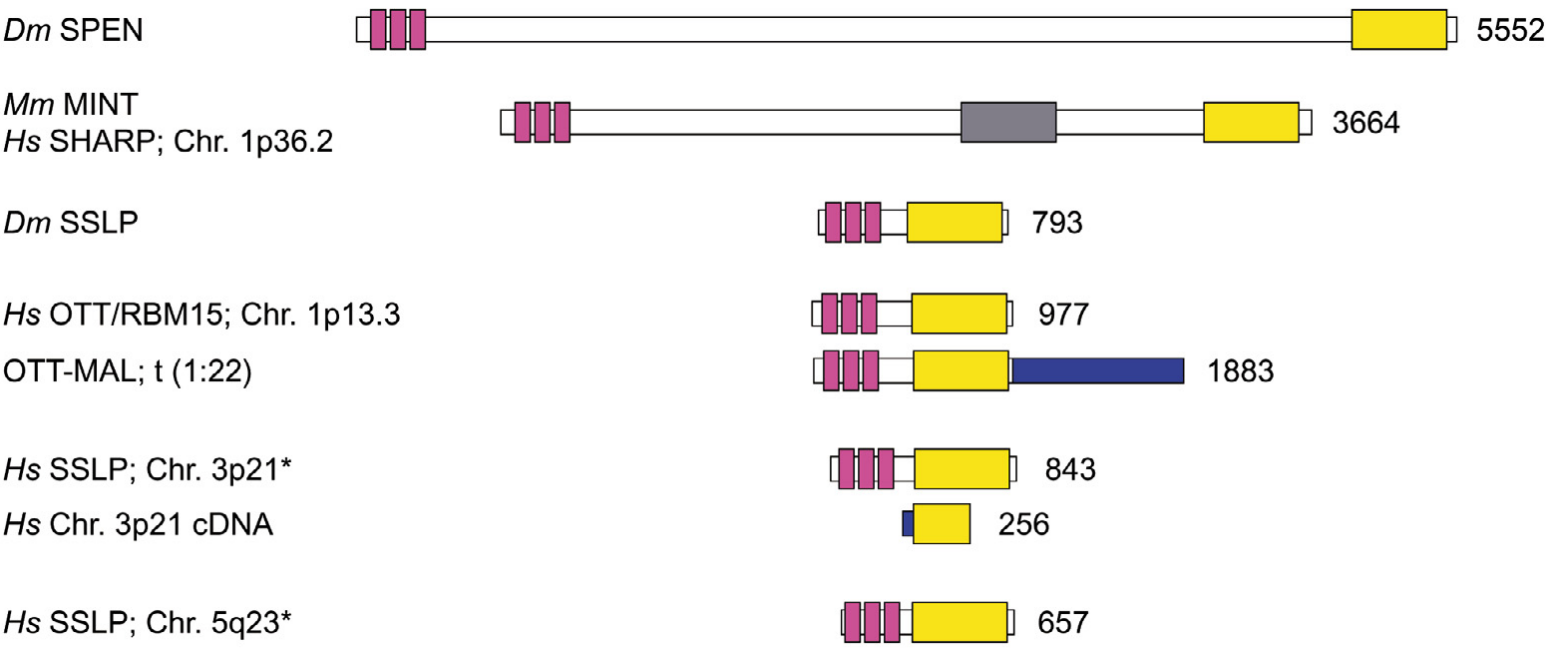
**Human *spen*-related genes**. The figure shows all *Drosophila *and human related sequences putatively encoding polypeptides with three RNP-type RNA binding motifs at the N terminus (purple boxes), and the SPOC domain at the C terminus (yellow boxes). The gray box on Hs SHARP indicates the region that contains motifs required for the interaction of SHARP/MINT with Msx-2 (residues 2070 to 2394 [14]), nuclear receptors (residues 2201 to 2707 [15]), and RBP-Jκ (residues 2803 to 2817 [17], and 2638 to 2777 [18]). The expected sizes for each peptide is shown on the right, while names and chromosomal localization in humans is on the left. Asterisks indicate that the peptides have been predicted from the genomic sequence, either because there are no known full length ESTs corresponding to the genomic regions analyzed (case for Hs SSLP at 3p21), or because there are no reported ESTs at all (Hs SSLP at 5q23). In the case of the SSLP at 5q23, there are also stop codons in frame with the putative ORF, so it is likely that this sequence represents a pseudogene. Accession numbers for the sequences likely to represent full length cDNAs are: AAF13218 (Dm SPEN), NP_055816 (Hs SHARP), AAF59160 (Dm SSLP), NP_073605 (OTT/RBM15), and CAC38829 (OTT-MAL fusion). The putative full length ORF for the Hs SSLP at 3p21 was predicted using GENESCAN [54] on the genomic sequence AC092037. The truncated cDNA arising from this gene is found under AAA72367 or NP_037418. The Hs SSLP at 5q23 was predicted with GENESCAN from the genomic segment AC005915, and the assembly was completed with ENSP322787, a predicted peptide from the Ensembl Database [42].

An influential role for *spen *in mechanisms of intercellular adhesion and/or cytoskeletal rearrangements may also be relevant to understanding its suggestive role in human cancer. The search of public human sequence resources [46,47] reveals one *spen *ortholog (SHARP), and three putative Short Spen-like Protein (SSLP) orthologs in the human genome (Figure 10). At least one of these genes (OTT/RBM15) is involved in a recurrent translocation detected in acute megakaryocitic leukemia [48,49], and a potentially aberrant transcript for another human SSLP ortholog at 3p21 has been identified in cDNA isolated from human cancer cells (Figure 10). Despite the presence of common domains, the functional relationship between large and small Spen-related polypeptides is still unknown. It is plausible that in *Drosophila*, SSLP might rescue some of the functions of *spen *during early embryonic development, as evidenced by the incomplete penetrance of phenotypes seen in *spen *maternal and zygotic mutant embryos. Complementation at this level has been suggested by others to explain the incomplete penetrance of *spen *mutations in wing discs [13], although it should be noted that the region required for Spen to interact with transcription factors such as Msx-2 or nuclear receptors, is apparently missing in SSLP proteins. Therefore, the potential redundancy of Spen and SSLP will have to be determined.

## Conclusions

**Figure 11:**
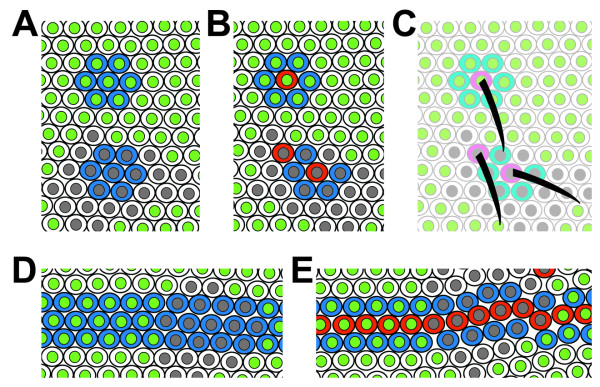
**A mechanistic model for Spen function**. The cartoon illustrates our conclusions to explain the defects seen in the presence of *spen *mutant clones. Wild type or heterozygote cells are depicted with green nuclei, and *spen *mutant cells with gray nuclei. The appearance of *spen *mutant cells in fields that will give rise to specific structures such as bristles or veins would imply the lack of an instructive signal to remain in place during growth of the disc. This will finally result in a progressive mis-localization of cells, ultimately leading to the abnormal positioning of structures after development is completed. Such a model would explain that, in some cases as in the proneural clusters, a change of fate is generated because negative instructive signals that depend on cell to cell contact are lost, resulting in the formation of two sensory organ precursor (SOP) cells within the same pro neural cluster (shown as red cells in B and C). The same situation may occur in the formation of wing veins, where similar Notch dependent regulatory mechanisms take place (E). Loss of or veins (or bristles) may occur when vein-forming cells move into intervein regions after their commitment has taken place, therefore leaving a hole where they should have been, which has been filled with cells that are unable to form vein at that spot.

We have shown that the function of the *spen *gene is essential for all stages of development. The experimental evidence indicates that Spen participates in processes that regulate planar cell polarity and may influence cytoskeletal organization, and its loss results in specific phenotypes that can not be solely explained by defects in a specific signaling pathway. In order to unify our observations with those previously reported by others, we would like to propose that the function of Spen is necessary for the maintenance of correct cell positioning during growth, ensuring that structures that are determined early during development are correctly positioned in the adult (Figure 11). As cells are determined early during development to become part of a specific structure, their position has to be maintained during growth according to a pre-established pattern. If cells were unable to maintain their position, we would expect phenotypes similar to those obtained in *spen *mutant clones. Structures would be misplaced, and in some cases would be absent if the cells that were predetermined to adopt a specific fate fall within "forbidden" positions (Figure 11). This mechanistic model could explain why *spen *interacts genetically with signaling pathways that require and/or specify precise spatial organization during metazoan development.

## Methods

### *Drosophila *stocks

All flies were grown at 21°C in standard medium, and were obtained from the Bloomington Stock Center, unless indicated otherwise. The stocks {*y*, *w; spen*^*poc*361^, *ck, FRT*^40*A*^*/In(2LR)O, Cy*}, {*MS1096-GAL4; spen*^*poc*361^, *ck, FRT*^40*A*^*/In(2LR)O, Cy*, *wg*-*lacZ*}, {*MS1096-GAL4; spen*^*poc*231^, *FRT *^40*A*^*/In(2LR)O*, *Cy*, *wg*-*lacZ*}, {*MS1096-GAL4; ck, FRT*^40*A*^*/In(2LR)O, Cy*, *wg-lacZ*}, {*MS1096-GAL4; 2 piM FRT*^40*A*^/ *In(2LR)O*, *Cy*, *wg-lacZ*}, {*ck FRT*^40*A*^/ *In(2LR)O*, *Cy; dpp*^*DISC*^-*GAL4/TM6B*}, {*spen*^*poc*361^, *ck FRT *^40*A*^*/In(2LR)O, Cy; dpp*^*DISC*^-*GAL4/TM6B*}, {*spen*^*poc*361^, *FRT*^40*A*^*/In(2LR)O*, *Cy; dpp*^*DISC*^-*GAL4/TM6B*}, {*spen*^*poc*231^, *FRT*^40*A*^*/In(2LR)O*, *Cy; dpp*^*DISC*^-*GAL4/TM6B*}, and {*y*, *w; GFP(2L), FRT*^40*A*^*; UAS-Flp1/TM6B*} were generated with the following lines: {*spen*^*poc*231^*/In(2LR)O*, *Cy*, *wg-lacZ*}, {*spen*^*poc*361^*/In(2LR)O*, *Cy*, *wg-lacZ*} [3], {*ck, FRT*^40*A *^*/In(2LR)O*, *Cy*} (obtained through recombination from the line {*S*^*X*155^, *ck, FRT *^40*A*^*/In(2LR)O*, *Cy*} [31], {w*; P{*w*^+*mC *^= *UAS-Flp1.D*}*JD2/TM3*, *Sb*^1^}, {*w*^1118^*; P*{*w*^+*mC *^= *Ubi-GFP(S65T)nls*}*2L P*{*ry*^+*t*7.2 ^= *neoFRT*^40*A*^}*/In(2LR)O*, *Cy*}, {*w*^1118^*; P*{*w*^+*mC *^= *piM*}*21C P*{*w*^+*mC *^= *piM}36F P{ry*^+*t*7.2 ^= *neoFRT*^40*A*^*}, {w*^1118^*; +; dpp*^*DISC*^*-GAL4/In(3LR)TM6B*} [28], and {*MS1096-GAL4*^*X*^} [22]. The line {*hsFlp1; M(2)36F, FRT*^40*A*^} was generated with {*hsFlp1; noc*^*Sco*^*/In(2LR)O, Cy*}, {*y*, *w; FRT*^40*A*^}, and {*M(2)36F/SM5*}. Other stocks used were {*ck*^14^*/In(2LR)O, Cy*}, {*ck*^16^*/ In(2LR)O, Cy*}, and y, w*; P{*w*^+*mC *^= *UAS-GFP::lacZ.nls*}*15:3*.

The *ovo*^D ^technique [50] was used to generate maternal germline clones as previously described [3]. These females were crossed to {*Df (2L)TE21A/In(2LR)O, Cy, wg-lacZ*} males, that carry a deletion spanning the *spen *locus, to obtain maternal and zygotic mutant embryos that were collected at 25°C.

### Immunodetection

To preserve GFP fluorescence, third instar imaginal discs were collected in cold PBS and fixed for 10–20 minutes on ice with methanol free 10% formaldehyde (Polysciences, Inc, Warrington, PA). After washing thoroughly with PBT (PBS with 0.1% Tween 20), and preincubating in the same buffer containing 10% Bovine Serum Albumin (BSA), the fixed discs were incubated with antibodies in PBT with 1% BSA. The mouse anti *Drosophila *Delta 594-9B monoclonal antibody (MAb) [51] was used purified at a concentration of 1:1000. The 22C10 [52], and anti Cut 2B10 [53] MAbs were obtained from the Developmental Studies Hybridoma Bank (DSHB, University of Iowa Department of Biological Sciences, Iowa City, IA), and used as recommended. The E-Cadherin rat MAb [54] was used at a 1:20 dilution. For fluorescent detection, FITC or Cy3-conjugated donkey anti mouse, or goat anti rat (Jackson Immunoresearch, West Grove, PA) were used. Discs were whole-mounted in mounting medium (Vector Laboratories, Burlingame, CA). Fluorescent images were captured with a Spot Digital Camera (Diagnostic Instruments, Inc, Sterling Heights, MI) using a Nikon Microphot-FXA microscope. Confocal images were acquired on a Leica TCS SP2 confocal microscope (Mannheim, Germany).

The embryonic expression of the neuron-specific marker Elav was immunodetected as described [55] with the mAb 9F8A9 [56] obtained from DSHB, and was used as a culture supernatant at 1:100, followed by incubation with a biotinylated goat anti-mouse (Jackson Immunoresearch, West Grove, PA), and a streptavidin-horse radish peroxidase (HRP) conjugate (DAKO, Carpinteria, CA). Similarly, β-galactosidase (LacZ) was detected with a rabbit antiserum (Cappel-ICN Biomedicals, Irvine, CA), at a 1:100 dilution, followed by a biotinylated goat anti-rabbit as above. Peroxidase activity was detected with the Immunopure Metal Enhanced DAB Substrate Kit (Pierce Biotechnology, Rockford, IL).

### Adult structures

Adult flies were collected in 70% ethanol, and stored in isopropanol. Wings were detached from the dehydrated adults and mounted with DPX (Fluka, Buchs, Switzerland). Notums were dissected, embedded in Lactic Acid: Hoyers (1:2) [57], and photographed in the same medium after clearing (usually 24 hours) using the equipment described above.

### Computer aided sequence analysis

Human genomic, and human and *Drosophila *cDNA sequences were retrieved from the Ensembl Genome Server [46], and from databases at the National Center for Biotechnology Information [47]. Sequence searches were performed using BLAST [58]. Composite multiple alignments were performed with MACAW [59] and Clustal X [60]. Genomic DNA sequences coding for putative *spen *related cDNAs in the human genome were analyzed by using GENESCAN [61].

## Abbreviations used

DAB: Di amino benzidine

GFP: Green Fluorescent Protein

HRP: Horseradish peroxidase

LacZ: β-galactosidase

MAb: Monoclonal antibody

SSLP: Short Spen-like protein

## Authors' contributions

KM performed the experiments shown in Figure 9, contributed to the generation and analysis of maternal and zygotic *spen *mutant embryos, and examined the expression of cell adhesion and polarity markers in *spen *mutant clones in wing discs, most of which are results not shown. She also actively participated in the writing and the elaboration of the conclusions of this work. AT did the rest.
